# Health-Plan and Employer-Based Wellness Programs to Reduce Diabetes Risk: The Kaiser Permanente Northern California NEXT-D Study

**DOI:** 10.5888/pcd10.120146

**Published:** 2013-01-31

**Authors:** Julie A. Schmittdiel, Susan D. Brown, Romain Neugebauer, Sara R. Adams, Alyce S. Adams, Deanne Wiley, Assiamira Ferrara

**Affiliations:** Author Affiliations: Susan D. Brown, Romain Neugebauer, Sara R. Adams, Alyce S. Adams, Deanne Wiley, Assiamira Ferrara, Kaiser Permanente Northern California Division of Research, Oakland, California.

## Abstract

Primary prevention of diabetes is increasingly recognized by both health plans and employers as an important strategy to improve the health of insured populations. As a part of the Natural Experiments in Translation for Diabetes (NEXT-D) network, the Kaiser Permanente Northern California (KPNC) Division of Research is assessing the effectiveness of 2 health plan-initiated programs to prevent the onset of diabetes in patients at high risk. The first study evaluates a telephonic health-coaching program that provides counseling on healthful eating, active living, and weight loss to KPNC members. The second evaluation examines a postpartum glucose screening and educational diabetes prevention program for women with gestational diabetes mellitus that KPNC implemented in 2006. Identifying effective approaches to preventing diabetes will be of value to health care systems, policy makers, and public health officials seeking to understand the roles systems and employers can play in preventing chronic illness.

## Introduction

Population approaches to improving health care and outcomes for patients with diabetes have been widely implemented by health plans and integrated delivery systems (1–4). Diabetes prevention is also increasingly emphasized by employers, who are major purchasers of health care insurance. Most companies with 50 or more employees offer worksite wellness programs (5), and nearly half of companies with more than 750 employees offer health risk assessment and screening (6). As awareness of diabetes risk grows in the employer/purchaser community, health plans and delivery systems are also adopting an active role in health promotion (7,8), with most offering primary prevention services such as health education and lifestyle programs (9–11). Efforts by health plans and employers to identify populations at high risk for developing diabetes and to focus prevention efforts on these populations are a promising strategy for reducing the incidence of the disease.

Health coaching to encourage healthy lifestyle choices (12–15) is a population-based approach to wellness and diabetes prevention that is being explored by health plans and purchasers. Health coaching, often via telephone, uses nonphysician health care providers to give patients the support, information, and skills required to improve self-efficacy and healthy behaviors. Health coaching may improve physical health status and healthy lifestyle behaviors (12–15) and may particularly benefit patients with prediabetes (16), who can benefit from coaching on lifestyle issues critical to diabetes prevention (17).

Women who have had 1 or more pregnancies marked by gestational diabetes mellitus (GDM) are another key group of health plan enrollees at heightened risk for diabetes (18). As the incidence of GDM rises, health plans are more often implementing guidelines to improve management of GDM during pregnancy and to encourage postpartum glucose screening for early identification and treatment (18,19) of those women who develop diabetes as well as promoting prevention strategies for those with prediabetes (20). As with other types of programs designed to decrease the risk of diabetes in high-risk patients, the effectiveness of these resource-intensive programs when broadly implemented in real-world clinical populations remains a question of interest to health plans, purchasers, and other stakeholders.

Kaiser Permanente Northern California (KPNC) is an integrated medical delivery system covering an enrolled population of 3.3 million members. KPNC provides numerous wellness and prevention programs to members, many of which encourage participation through partnerships with purchasers. The Natural Experiments in Translation for Diabetes (NEXT-D) study provides a unique opportunity for KPNC researchers and operations leaders, partnering together using participatory research principles (21), to evaluate the effect of health-plan–based diabetes prevention programs on populations at high risk for developing the disease. We outline our plans to use quasi-experimental methods to evaluate 2 such programs focused on diabetes prevention: a Wellness Coaching program and a postpartum glucose screening and diabetes prevention program for women with GDM.

## Wellness Coaching Program Evaluation

KPNC’s Regional Health Education Department launched a telephonic Wellness Coaching Center in January 2010 targeted at members interested in healthy behaviors and employers who want to encourage healthy lifestyles among their employees. Wellness Coaching is designed to help members set and reach goals in 5 key areas (healthful eating, physical activity, weight management, smoking cessation, and stress management) to reduce health risk and increase health-related quality of life. Coaches are trained in motivational interviewing (12), a patient-centered behavioral health approach that assists patients in addressing self-management and multiple health risks and behaviors. This approach to health coaching has been effective in improving patient physical and mental health status (12). A typical coaching engagement consists of 1 initial session (30 minutes) and up to 3 short (5 to 15 minutes) follow-up contacts. Wellness Coaching is available to all KPNC members, and the program recruits participants through mechanisms that include partnerships with employers and direct risk assessment and outreach.

Our evaluation of the Wellness Coaching program has 3 phases. Phases 1 and 2 focus on the 1,427 patients who participated in Wellness Coaching from January 1 through August 23, 2011. Patients who participated in coaching during this period were predominantly female (80%) and had an average body mass index (BMI) of 33.1 kg/m^2^. Approximately one-half of these patients (47%) were white.

Phase 1 consists of a patient survey designed to examine patient-centered experiences with the program and predictors of self-perceived coaching success. This cross-sectional survey, which was designed by the research team using validated metrics, focuses on 4 domains: patient satisfaction, reasons for using the wellness coaching program, self-reported changes in healthy behaviors, and patient engagement in their health care. Phase 2 of the evaluation will use an interrupted time series with concurrent control groups (22) to assess the effect of Wellness Coaching on levels of BMI, systolic blood pressure, and low-density lipoprotein cholesterol (LDL-c) levels. If phases 1 and 2 suggest that Wellness Coaching has a positive effect on outcomes, phase 3 will use a randomized trial to compare the effectiveness of 3 outreach methods (letters, interactive voice–response telephone messages, and secure e-mail) on increasing rates of Wellness Coaching participation among approximately 30,000 patients with impaired fasting glucose (IFG).

## Gestational Diabetes Program Evaluation

Within KPNC, the Regional Perinatal Service Center offers supplemental care via telephone counseling for women with pregnancies at high risk for adverse outcomes (such as preterm birth), including pregnancies complicated by GDM. Referral to the centers has been associated with decreased risk of macrosomia (excessive birth weight) and increased postpartum screening for diabetes (23). Beginning in 2006, the center has used a step-wise approach to ensure that all patients have a glucose test (standard oral glucose tolerance test [OGTT]) and appropriate educational and referral follow-up if postpartum glucose levels are elevated (diagnostic for IFG, impaired glucose tolerance, or diabetes). These increases in screening and the shift to the more sensitive OGTT should lead to greater detection of prediabetes and earlier detection of type 2 diabetes. We will evaluate whether the incidence of diabetes is decreasing because of these earlier detection and prevention efforts and because of treatment efforts launched after detection. We will compare the cohort of women with GDM who gave birth during 2001 through 2006 with the cohort who gave birth during 2006 through 2010 for subsequent diabetes incidence. For each cohort, follow-up begins at childbirth and continues until the diagnosis of diabetes, to the end of the study period (5 years follow-up for each person), or censoring because of leaving the health plan.

## Real-World Opportunity for Natural Experiments

Health systems, employers, and health plan purchasers recognize the urgency of determining whether their population-oriented infrastructure can be adapted to address primary prevention of chronic conditions such as diabetes. Given the large numbers of people at increased risk for these conditions, efficient approaches are needed to identify and support patients and providers in effecting lifestyle changes. Evaluating these approaches will be useful to policy makers dealing with questions of benefit designs and to public health officials seeking to understand the roles that health systems and employers can play in preventing chronic disease. Health care policy initiatives emphasizing the patient-centered medical home and accountable care organizations (24,25) are being promoted as ways to enhance the integration of US health care delivery. Systems with high levels of integration such as KPNC offer real-world opportunities for natural experiments to assess the effect of health-plan and employer-based prevention and wellness programs on population health within this context.
